# Verlorene Lebensjahre: Bekanntes und Neues zur Methodik am Beispiel der häufigsten Todesursachen in Deutschland

**DOI:** 10.1007/s00103-021-03424-8

**Published:** 2021-10-01

**Authors:** Joachim Hübner, Johann Mattutat, Alexander Katalinic

**Affiliations:** 1 Institut für Sozialmedizin und Epidemiologie, Universität zu Lübeck, Ratzeburger Allee 160, 23562 Lübeck, Deutschland; 2 Institut für Krebsepidemiologie, Universität zu Lübeck, Lübeck, Deutschland

**Keywords:** Mortalität, Todesursachen, Verlorene Lebensjahre, Methodik, Gesundheitssystemplanung, Mortality, Causes of death, Years of life lost, Methods, Health planning

## Abstract

**Hintergrund:**

Verlorene Lebensjahre (Years of Life Lost, YLL) sind ein aussagekräftiger, in Deutschland jedoch wenig verwendeter Indikator für die Relevanz von Todesursachen. Es existieren zahlreiche Methoden, mit denen YLL berechnet werden können.

**Ziel der Arbeit:**

Es werden prototypische Methoden zur Berechnung von YLL vorgestellt und kritisch eingeordnet. Auf dieser Basis wird eine verbesserte Methode vorgeschlagen, die auf der Nutzung von todesursachenbereinigten Sterbetafeln (Cause-Elimination Life Tables, CELT) beruht.

**Methoden:**

Etablierte Methoden und die hier vorgeschlagene Modifikation werden auf die Sterblichkeit in Deutschland 2018 angewandt. Veränderungen gegenüber 1998 werden anhand der modifizierten Methode dargestellt.

**Ergebnisse:**

Während nach der Zahl der Sterbefälle Herz-Kreislauf-Erkrankungen im Jahr 2018 die bedeutendste Todesursache waren, war Krebs für die meisten YLL verantwortlich. Unterschiedliche Methoden zur Berechnung der YLL führen zu deutlich abweichenden Rängen bei den weniger bedeutsamen Todesursachen. YLL auf Basis von allgemeinen Sterbetafeln unterschätzen die YLL auf Basis der todesursachenbereinigten Sterbetafeln um bis zu 18,4 % (Herz-Kreislauf-Erkrankungen). Gemessen an den CELT-basierten YLL waren im Jahr 1998 Herz-Kreislauf-Erkrankungen die bedeutsamste Todesursache.

**Diskussion:**

Die Berechnung von YLL auf der Basis von todesursachenbereinigten Sterbetafeln vermeidet Inkonsistenzen etablierter Methoden und führt zu relevant abweichenden Ergebnissen. Besonderheiten der vorgeschlagenen Methode (Verstoß gegen das Egalitätsprinzip, fehlende Additivität) beeinträchtigen ihren Nutzen als Instrument zur Steuerung der Gesundheitsversorgung nicht.

**Zusatzmaterial online:**

Zusätzliche Informationen sind in der Online-Version dieses Artikels (10.1007/s00103-021-03424-8) enthalten.

## Abstract (en)

**Background:**

Years of life lost (YLL) is a meaningful indicator of the relevance of causes of death, although it is rarely used in Germany. Numerous methods have been developed to calculate YLL.

**Objective:**

Prototypical methods for calculating YLL are presented and critically appraised. An improved method is proposed that is based on cause-elimination life tables (CELTs).

**Methods:**

The different methods are applied to deaths in Germany in 2018. Changes in comparison to 1998 are presented using the modified method.

**Results:**

While in 2018 cardiovascular diseases were the leading cause of death by number of cases, cancer was responsible for most YLL. Different methods of calculating YLL lead to divergent ranks for less frequent causes of death. YLL on the basis of general life tables underestimate CELT-based YLL by up to 18.4% (cardiovascular diseases). Measured by CELT-based YLL, cardiovascular diseases were the most important cause of death in 1998.

**Conclusion:**

The calculation of YLL on the basis of CELTs avoids inconsistencies of established methods and leads to relevantly different results. Characteristics of the proposed method (violation of the egalitarian principle, lack of additivity) do not affect its usefulness as a tool for health planning.

## Einleitung

Eine rationale Steuerung der Gesundheitsversorgung ist auf Indikatoren angewiesen, die ein realistisches Bild von der Krankheitslast in der Bevölkerung vermitteln [1]. Je größer der Schaden ist, den eine Erkrankung für die Lebensqualität und/oder die Lebenserwartung der Menschen verursacht, desto eher ist gerechtfertigt, gesellschaftliche Ressourcen in ihre Bekämpfung durch Prävention, Therapie und Forschung zu investieren. Besondere Aufmerksamkeit erfährt regelmäßig die krankheitsspezifische Sterblichkeit. Entsprechende Fallzahlen, rohe und altersstandardisierte Raten zählen zu den epidemiologischen Standardinformationen in der medizinischen Fachliteratur. Ein Ranking der häufigsten Todesursachen ist Kernbestandteil der Gesundheitsberichterstattung des Bundes, wird gerne in den Medien thematisiert und beeinflusst gesundheitspolitische Weichenstellungen. So wurde etwa die Tatsache, dass Krebs die zweithäufigste Todesursache in Deutschland ist, als übergeordnete Motivation für die 2019 ausgerufene Nationale Dekade gegen Krebs angeführt [2]. Das besondere Interesse an der Mortalität kann damit erklärt werden, dass der Tod eines Menschen nicht nur die schwerstmögliche Folge einer Erkrankung ist. Er ist im Vergleich zur Morbidität (Inzidenz und Prävalenz) und Lebensqualität auch weniger anfällig für unterschiedliche Definitionen und Verfahren der Operationalisierung.

Allerdings kann die Fokussierung auf die Häufigkeit einer Todesursache zu kontraintuitiven Schlüssen führen. Für die meisten Menschen macht es einen großen Unterschied, ob jemand jung, etwa im Alter von 35 Jahren, oder im fortgeschrittenen Lebensalter, etwa mit 70 Jahren, stirbt [3]. Respektiert man diese Intuition, ist aus der Perspektive der Gesundheitssystemplanung danach zu differenzieren, ob eine Erkrankung typischerweise erst im hohen Alter oder bereits in jüngeren Jahren todesursächlich wird. Dem trägt das Konzept der verlorenen Lebensjahre (Years of Life Lost, YLL) Rechnung. In diesem Konzept werden nicht einfach Todesfälle gezählt, sondern es wird jeweils hypothetisch festgestellt, wie viel Lebenszeit durch diese Todesfälle verloren geht. Sieht man von methodischen Sonderlösungen ab, die teilweise für den Tod von Kindern in Betracht gezogen werden, ist den Methoden gemeinsam, dass der Tod eines jungen Menschen ein größeres Gewicht erhält als der Tod eines älteren. Das Konzept der YLL ist international etabliert, findet, von einigen Studien abgesehen [1, 4–6], in Deutschland jedoch nur wenig Beachtung. Ein Grund dafür mögen die Unsicherheiten sein, wie die durch den Tod abgeschnittenen Lebensjahre sinnvoll zu quantifizieren sind.

In diesem Beitrag werden unterschiedliche Berechnungsweisen für YLL vorgestellt und es wird eine Modifikation vorgeschlagen, die sich insbesondere für die Steuerung der Gesundheitsversorgung eignet. Die unterschiedlichen Methoden werden auf die Sterblichkeit in Deutschland 2018 angewandt. Anhand der modifizierten Methode werden Veränderungen der Sterblichkeit im Vergleich zu 1998 dargestellt.

### Etablierte Methoden

Eine einfache Methode zur Berechnung verlorener Lebensjahre besteht darin, für jeden Todesfall der interessierenden Ursache die Differenz bis zu einem festen Referenzalter festzustellen. Die interaktive Onlinedatenbank der Gesundheitsberichterstattung des Bundes bietet als Referenzalter 65 und 70 Jahre an [7]. Verstirbt also jemand im Alter von 60 Jahren an Krebs, werden 5 bzw. 10 Jahre als verloren gezählt. Todesfälle jenseits des Referenzalters bleiben unberücksichtigt. Werden die so ermittelten Werte für alle Krebstodesfälle aufsummiert, ergibt sich die Gesamtzahl aller „potenziell verlorenen Lebensjahre“ (Potenzial Years of Life Lost, PYLL) durch Krebs. Erkrankungen wie Morbus Parkinson, die typischerweise erst im hohen Lebensalter zum Tode führen, erhalten bei dieser Methode ein geringes Gewicht.

Diese Konsequenz wird abgemildert, wenn als Referenzalter nicht ein Wert in der Nähe des Renteneintrittsalters dient, sondern die Lebenserwartung eines Menschen bei Geburt [8]. Die aktuellen Lebenserwartungen bei Geburt betragen in Deutschland 83,4 Jahre für Mädchen und 78,6 Jahre für Jungen (Durchschnitt 2017–2019; [7]). Durch den Tod einer 60-jährigen Frau gehen danach ca. 23,4 Lebensjahre, durch den Tod eines 60-jährigen Mannes ca. 18,6 Lebensjahre verloren. Problematisch ist diese Methode, wenn YLL unterschiedlicher Länder miteinander verglichen werden sollen. Der Tod eines Menschen in einem Land mit relativ geringer Lebenserwartung bedeutet dann einen geringeren Verlust als der Tod eines gleich alten Menschen in einem Land mit höherer Lebenserwartung. Darin wird ein Verstoß gegen den egalitären Anspruch der vergleichenden Gesundheitsberichterstattung gesehen („treating like events as like“; [9]). Diesem Problem kann begegnet werden, indem länderübergreifend einheitliche Lebenserwartungen angenommen werden, z. B. die aktuell höchsten Lebenserwartungen: 87,6 Jahre für Frauen (Singapur) bzw. 82,1 Jahre für Männer (Schweiz; [10]).

Ungeachtet dessen haben sämtliche Methoden, die fixe Lebensspannen, ob willkürlich gewählt oder empirisch begründet, zugrunde legen, eine Schwachstelle. Sie ignorieren, dass jeder Tod eines Menschen, egal in welchem Alter, Lebenszeit abschneidet [9]. Folgerichtig sind demgegenüber Methoden, die beim Tod eines Menschen im Alter von x Jahren so viele Lebensjahre als verloren zählen, wie es der statistischen Lebenserwartung eines Menschen im Alter von x Jahren entspricht. So berechnete YLL werden auch Expected Years of Life Lost (EYLL) genannt [11, 12]. Die notwendigen Informationen werden Sterbetafeln entnommen, die auf der Basis von altersspezifischen Sterbewahrscheinlichkeiten errechnet werden (daher auch „Sterbetafelmethode“). Beispielhaft weist die deutsche Sterbetafel für eine 60-jährige Person eine Lebenswartung von weiteren 25,4 Jahren (weiblich) bzw. 21,8 Jahren (männlich) aus [7], die bei Berechnung der EYLL berücksichtigt würden. Man beachte, dass die resultierenden hypothetischen Sterbealter (85,4 bzw. 81,8 Jahre) höher liegen als die Lebenserwartungen bei Geburt, da das erreichte Lebensalter von 60 Jahren die Möglichkeit eines früheren Todes ausschließt, was sich erhöhend auf das statistisch zu erwartende Sterbealter auswirkt.

Die Sterbetafelmethode wird auch im internationalen Global-Burden-of-Disease-(GBD-)Projekt angewandt. Dem erwähnten Egalitätsprinzip entsprechend werden im GBD-Projekt allerdings keine regionalen Sterbetafeln verwendet, sondern eine einheitliche Matrix. Sie bildet nicht die Sterblichkeit in einem realen Referenzstaat ab, sondern einen idealisierten Standard. Dieser beruht auf Lebenserwartungen unter der hypothetischen Annahme besonders günstiger Umstände in jedem Lebensalter (weitgehende Kontrolle vermeidbarer Risiken und gute gesundheitliche Versorgung; [13]). Dazu werden die niedrigsten altersgruppenspezifischen Sterbewahrscheinlichkeiten aus unterschiedlichen Ländern kombiniert [14]. Dem Egalitätsprinzip folgend wird auch nicht zwischen den Geschlechtern differenziert. Ergebnis ist ein dynamischer Standard mit Lebenserwartungen, die in keinem Land der Welt tatsächlich erreicht werden. Auf solcher Grundlage berechnete YLL werden auch Standard Expected Years of Life Lost (SEYLL) genannt [15]. In der GBD-Studie 2017 beträgt die Lebenserwartung bei Geburt für Mädchen und Jungen 87,9 Jahre. Beim Tod einer 60-jährigen Person würden 29,3 Lebensjahre verloren gehen [10].

Bei den bislang vorgestellten Varianten der Berechnung hat jedes YLL denselben Zählwert – nämlich 1. In früheren GBD-Studien wurde von diesem Prinzip abgewichen [16]. In Anlehnung an Konzepte der Finanzmathematik wurde angenommen, dass Lebenszeitgewinne, die sich erst in ferner Zukunft realisieren, geringer zu bewerten sind als solche, die unmittelbar bevorstehen. Entsprechend wiegt der Verlust des jeweils nächsten Lebensjahres bei 10 Personen schwerer als der Verlust von 10 Lebensjahren durch den Tod eines Menschen, da in letzterem Fall der Schaden erst in durchschnittlich 5 Jahren eintritt. Ein typischer Zinssatz, mit dem die zukünftigen Lebenszeitgewinne „abdiskontiert“ werden, beträgt 3 % per anno [9]. In einem anderen, von der Nützlichkeitsethik des Utilitarismus beeinflussten Ansatz wurden Lebensjahre in Abhängigkeit vom Lebensalter unterschiedlich gewichtet. Aufgrund größerer Verantwortung für das Wohl der Gesellschaft erhielten Lebensjahre im jungen und mittleren Erwachsenenalter teilweise ein größeres Gewicht als Lebensjahre in der Kindheit und im höheren Alter [9]. Dieses Verfahren ist heute kaum noch akzeptanzfähig.

### Weiterentwicklung auf Basis ursachenbereinigter Sterbetafeln

Ein grundsätzliches Problem aller dargestellten Sterbetafelmethoden wird bislang nur vereinzelt thematisiert [5]. Geht es um die Frage, welchen Nutzen es in der Bevölkerung hätte, wenn eine Erkrankung als Todesursache eliminiert wird, ist es offenbar unstimmig, Lebenserwartungen aus allgemeinen Sterbetafeln heranzuziehen. Die Sterbewahrscheinlichkeiten, die dort zugrunde liegen, beruhen auf Sterbefällen jeder Ursache und sind folglich auch durch die zu eliminierende Indexerkrankung beeinflusst. Fallen Sterbefälle einer bestimmten Ursache in der Bevölkerung weg, erhöhen sich zwangsläufig die Lebenserwartungen bei Geburt und – wenn die eliminierte Sterblichkeit nicht auf das erste Lebensjahr beschränkt ist – auch die ferneren Lebenswartungen in anderen Altersstufen.

Sterbetafeln, die dies berücksichtigen, sind international als Cause-Elimination Life Tables (im Folgenden: CELT) bekannt. CELT werden für spezifische Todesursachen erstellt und geben Lebenserwartungen unter der Prämisse an, dass eine Erkrankung oder Erkrankungsgruppe als Todesursache eliminiert wird [17]. Die Differenz zwischen der CELT-basierten Lebenserwartung bei Geburt und der entsprechenden Lebenserwartung aus der allgemeinen Sterbetafel gibt darüber Auskunft, welche Auswirkung die Elimination einer Todesursache auf die Lebenserwartung jedes Einzelnen statistisch hätte [17–20]. In der Gesundheitsberichterstattung des Bundes ist dieser Indikator als „Gewinn an Lebenserwartung“ eingeführt [7]. Als Steuerungshilfe für das Gesundheitssystem ist er nur bedingt geeignet, da er – nicht anders als die Lebenswartungen aus den allgemeinen Sterbetafeln – unabhängig vom Altersaufbau der Bevölkerung ist. Zum Beispiel hängt die Auswirkung der aktuellen Coronapandemie auf die Lebenserwartung von den altersspezifischen Risiken und der Versorgungssituation ab. In 2 Bevölkerungen, in denen diese Bedingungen gleich sind, ändert sich auch die Lebenserwartung in gleichem Ausmaß. Ist in einer dieser beiden Bevölkerungen aber das Durchschnittsalter höher als in der anderen, sterben dort mehr Menschen und es geht mehr Lebenszeit verloren. Um die sterblichkeitsbezogene reale Krankheitslast abzubilden, ist es also folgerichtig, die CELT-basierten Lebenserwartungen auf die Sterbefälle in der jeweiligen Bevölkerung anzuwenden. Dies ist der Grundgedanke unseres methodischen Vorgehens.

## Methode

Wir haben die Konsequenzen der Anwendung unterschiedlicher Sterblichkeitsindikatoren auf Todesursachen in Deutschland untersucht. Betrachtet wurden die Zahl der Sterbefälle, die PYLL unter 65 Jahre (PYLL _<_ _65_) und YLL nach der Sterbetafelmethode (EYLL). Bei Letzterer verglichen wir die entsprechenden Kennzahlen auf Basis der regulären und ursachenbereinigten Sterbetafeln (konventionelle EYLL bzw. CELT-basierte YLL). Bei der Auswahl der zu vergleichenden Indikatoren wurde die Zahl der Sterbefälle als De-facto-Standard beim Ranking der bedeutsamsten Todesursachen gewählt. PYLL unter 65 Jahre wurde als extremes Gegenbeispiel gewählt. Das Sterbealter, das bei der Zahl der Sterbefälle überhaupt keine Rolle spielt, erhält dort im Vergleich aller geläufigen Indikatoren das größte Gewicht. YLL auf Basis der allgemeinen Sterbetafel wurden betrachtet, da die hier vorgeschlagene Berechnung verlorener Lebensjahre eine Modifikation dieser Methode ist, deren praktische Konsequenzen darzustellen sind.

Als Datenbasis dienten uns Sterbefälle des Jahres 2018 der GBD-Studie, abgerufen über die Onlinedatenbank des Institute for Health Metrics and Evaluation (IHME; [21]). Auf dem von uns gewählten zweiten hierarchischen Level sind 21 Ursachen definiert. Anders als in der amtlichen deutschen Todesursachenstatistik werden im GBD-Projekt nichtinformative (nicht korrekt oder nicht eindeutig codierte) Todesursachen („garbage codes“) mithilfe eines standardisierten Algorithmus umverteilt [22, 23].

Auch die Ursachenkategorien des GBD-Projekts weichen im Detail von der Systematik der ICD‑9 und ICD-10 (Internationale statistische Klassifikation der Krankheiten und verwandter Gesundheitsprobleme [International Statistical Classification of Diseases and Related Health Problems], 9. bzw. 10. Revision) ab [24]. Beispielsweise wird die Karditis durch Viren (ICD-10 B33.2) nicht den Infektionskrankheiten, sondern den Herz-Kreislauf-Erkrankungen (B.2 Cardiovascular Diseases) zugeordnet, die juvenile Arthritis bei Crohn-Krankheit – in der ICD-Systematik eine Krankheit des Muskel-Skelett-Systems (ICD-10 M09.1) – zählt im GBD-Projekt als gastroenterologische Erkrankung (B.4 Digestive Diseases). Eine Synopse der GBD-Klassifikationen und der ICD-9- und ICD-10-Codes ist im Onlinematerial wiedergegeben (Anhang 1). Sterbefälle wurden für 21 verfügbare Altersgruppen (0-< 1 Jahr, 1‑< 5 Jahre, 5‑< 10 Jahre, 10-< 15 Jahre, …, ≥ 95 Jahre) nach Geschlecht extrahiert. Die zugrunde liegenden Bevölkerungszahlen wurden der GENESIS-Online-Datenbank des Statistischen Bundesamtes entnommen [25].

Auf dieser Grundlage ermittelten wir für beide Geschlechter altersspezifische Sterbewahrscheinlichkeiten und berechneten daraus je eine allgemeine Sterbetafel für Frauen und Männer nach der üblichen Methodik für Periodensterbetafeln [26]. Außerdem berechneten wir je Geschlecht für jede Ursache eine spezifische, um diese Ursache bereinigte Sterbetafel. Das Verfahren ist ausführlich bei Arias et al. beschrieben [17]. Kurz gefasst besteht es darin, dass von der beobachteten altersgruppenspezifischen Sterbewahrscheinlichkeit die jeweilige ursachenspezifische Sterbewahrscheinlichkeit subtrahiert und ein Korrekturterm addiert wird. Der Korrekturterm ist erforderlich, da Personen, die wegen hypothetischer Elimination einer Todesursache länger leben, in der gewonnenen Lebenszeit dem Risiko eines Todes infolge anderer Ursachen ausgesetzt sind. Dabei wird wiederum davon ausgegangen, dass dieses individuelle Risiko dem allgemeinen Sterberisiko entspricht, d. h., die Wahrscheinlichkeit für konkurrierende Todesursachen wird als unabhängig angesehen (Anhang 2 im Onlinematerial).

YLL nach Geschlecht wurden jeweils durch Multiplikation der Sterbefälle je Altersgruppe mit den altersspezifischen Lebenserwartungen aus der regulären bzw. CELT-basierten Sterbetafel berechnet. Zur Ermittlung der Gesamtzahl der YLL nach Todesursache wurden YLL aller Altersgruppen und beider Geschlechter aufsummiert. YLL auf der Basis von todesursachenbereinigten Sterbetafeln wurden außerdem für das Jahr 1998 berechnet. Auch insoweit verwendeten wir die Datenbank des GBD-Projekts und die GENESIS-Online-Datenbank des Statistischen Bundesamtes.

## Ergebnisse

**Figure 1:**
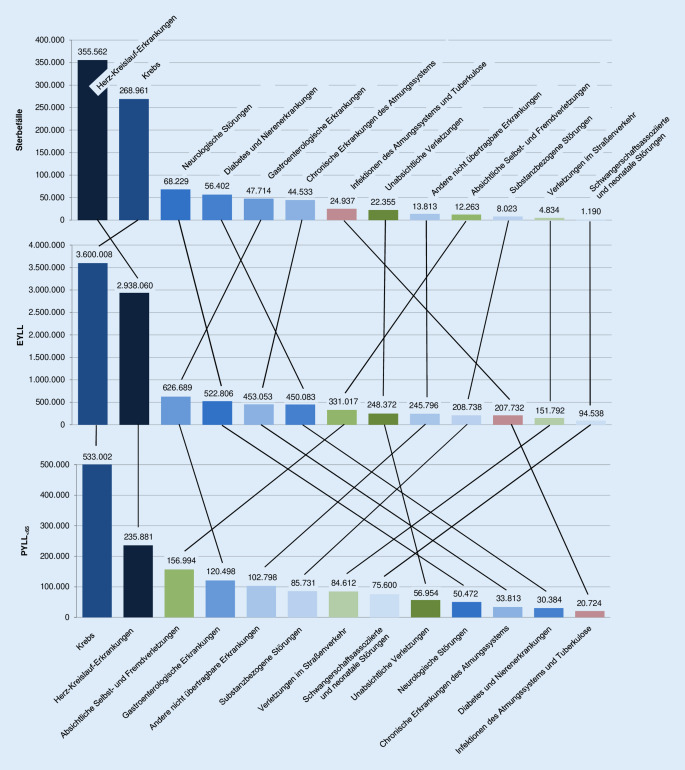

In Abb. 1 ist die vorzeitige ursachenspezifische Sterblichkeit in Deutschland im Jahr 2018 anhand der etablierten Indikatoren Zahl der Sterbefälle, PYLL _<_ _65_ und EYLL dargestellt. Berücksichtigt sind alle Ursachen, die nach einem der Indikatoren einen der ersten 10 Rangplätze belegen. Nach Zahl der Sterbefälle dominieren Herz-Kreislauf-Erkrankungen und Krebs die Mortalität. Gemessen an den EYLL gilt dies ebenfalls, wobei aber die Herz-Kreislauf-Erkrankungen hinter Krebs zurücktreten. Bei der Betrachtung der potenziell verlorenen Lebensjahre < 65 Jahre (PYLL _<_ _65_) rücken auch seltenere Todesursachen in den Fokus, die für einen relativ großen Anteil der Sterblichkeit in jüngeren Lebensjahren verantwortlich sind: substanzbezogene Störungen (Rang 6), Verletzungen im Straßenverkehr (Rang 7) sowie schwangerschaftsassoziierte und neonatale Störungen (Rang 8). Auch absichtliche Selbst- und Fremdverletzungen erhalten ein deutlich höheres Gewicht, als es der Häufigkeit der Todesursache entspricht (Rang 3 vs. Rang 10). Umgekehrt verlieren neurologische Störungen (Rang 10 vs. Rang 3) sowie Diabetes und Nierenerkrankungen stark an Bedeutung (Rang 12 vs. Rang 4). Die Ränge, die sich auf Basis der YLL nach Sterbetafelmethode ergeben, liegen oft zwischen denen, die sich auf Basis von Sterbefällen einerseits und potenziell verlorenen Lebensjahren (PYLL _<_ _65_) andererseits ergeben.

In der Tab. 1 werden YLL nach herkömmlicher Sterbetafelmethode (EYLL) mit YLL auf Basis von todesursachenbereinigten Sterbetafeln (CELT-basierte YLL) verglichen. Bei Betrachtung der absoluten Zahlen unterschätzen die EYLL durch Herz-Kreislauf-Erkrankungen und Krebs die korrigierten Ergebnisse um 18,4 % bzw. 10,5 %. Der Vergleich mit Abb. 1 zeigt, dass das Ausmaß der Unterschätzung generell umso größer ist, je häufiger die Todesursache ist. Daraus ergeben sich Änderungen bei der Rangfolge: Diabetes und Nierenerkrankungen rücken im Wechsel mit chronischen Erkrankungen der Atmungssystems von Rang 6 auf Rang 5 vor: Auf den Rängen 10 und 11 wechseln Infektionen des Atmungssystems und Tuberkulose mit substanzbezogenen Störungen die Position.

**Table 1:** 

Todesursache	EYLL auf Basis der regulären Sterbetafel (Rang)	CELT-basierte YLL (Rang)	Abweichung in % (EYLL vs. CELT-basierte YLL)
Krebs	3.600.008 (1)	4.020.225 (1)	−10,5
Herz-Kreislauf-Erkrankungen	2.938.060 (2)	3.601.007 (2)	−18,4
Gastroenterologische Erkrankungen	626.689 (3)	637.607 (3)	−1,7
Neurologische Störungen	522.806 (4)	541.512 (4)	−3,5
Chronische Erkrankungen des Atmungssystems	453.053 (5)	462.726 (6)	−2,1
Diabetes und Nierenerkrankungen	450.083 (6)	463.313 (5)	−2,9
Absichtliche Selbst- und Fremdverletzungen	331.017 (7)	332.486 (7)	−0,4
Unabsichtliche Verletzungen	248.372 (8)	250.500 (8)	−0,8
Andere nichtübertragbare Erkrankungen	245.769 (9)	246.711 (9)	−0,4
Substanzbezogene Störungen	208.738 (10)	209.437 (11)	−0,3
Infektionen des Atmungssystems und Tuberkulose	207.732 (11)	210.378 (10)	−1,3

*CELT* Cause-Elimination Life Tables

**Figure 2:**
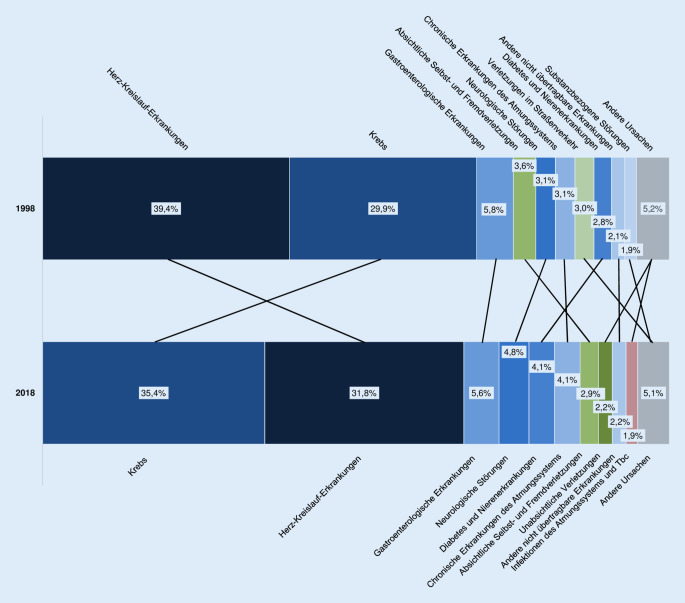

Abb. 2 stellt dar, in welchem Ausmaß sich die relative Bedeutung der 10 häufigsten Todesursachen, gemessen an den CELT-basierten YLL, von 1998 bis 2018 verändert hat. Im Gegensatz zu 2018 waren 1998 Herz-Kreislauf-Erkrankungen für den größten Verlust an Lebenszeit verantwortlich. Ebenfalls an Bedeutung verloren haben absichtliche Fremdverletzungen und Verletzungen im Straßenverkehr. Am stärksten an Bedeutung gewonnen haben neben Krebs Diabetes und Nierenerkrankungen sowie unabsichtliche Verletzungen.

## Diskussion

Wie gezeigt, messen unterschiedliche Sterblichkeitsindikatoren den Todesursachen eine unterschiedliche Relevanz zu. Am deutlichsten treten die Unterschiede beim Vergleich des Rankings nach Sterbefällen mit dem Ranking nach verlorenen Lebensjahren unter 65 Jahren (PYLL _<_ _65_) zutage. Rangzuweisungen nach YLL auf der Basis realistischerer Lebenserwartungen (z. B. Lebenserwartung bei Geburt oder nach Sterbetafelmetode) liegen typischerweise zwischen denen, die sich aus den erstgenannten Methoden ergeben. Beispielhaft haben die Todesursachen absichtliche Selbst- und Fremdverletzungen einerseits und Diabetes und Nierenerkrankungen andererseits nach EYLL ähnliche Relevanz. Nach Zahl der Todesfälle übersteigt die Bedeutung Letzterer die absichtlichen Selbst- und Fremdverletzungen um den Faktor 4,6 oder 6 Rangstufen. Nach PYLL _<_ _65_ sind umgekehrt die absichtlichen Selbst- und Fremdverletzungen weitaus bedeutsamer (um den Faktor 5,2 bzw. 9 Rangstufen).

Entscheidungen für oder gegen eines der konkurrierenden Konzepte sind nicht richtig oder falsch, sondern Ausdruck von Wertungen, die – wenn sie die Ressourcenverteilung im Gesundheitssystem beeinflussen sollen – einer ethischen Legitimation bedürfen. Indikatoren, die ausschließlich Lebensjahre im produktiven Lebensalter berücksichtigen, sind insoweit offensichtlich problematisch, da sie einen Zusammenhang zwischen der Behandlungswürdigkeit von Menschen und ihrem Nutzen für die Gesellschaft implizieren [27]. Unabhängig von der konkreten Berechnungsweise geraten letztlich aber alle Priorisierungsvorhaben, die YLL zum Maßstab nehmen, in potenziellen Konflikt mit dem Prinzip der Lebenswertindifferenz, wonach jedes Leben, ob jung oder alt, gleich wertvoll ist [28]. Das Prinzip wurde jüngst im Zusammenhang mit der Triage von intensivpflichtigen Patienten in der COVID-19-Pandemie thematisiert [29]. Im hier behandelten Kontext spricht die Indirektheit der Wirkung gegen die Annahme einer unzulässigen Altersdiskriminierung: Es wird nicht über konkrete Menschenleben entschieden, sondern es geht abstrakt um die Allokation von Mitteln zur Bekämpfung von Krankheiten oder anderen Todesursachen, die mit unterschiedlicher Häufigkeit in unterschiedlichen Lebensphasen auftreten.

Für das Konzept der YLL spricht, dass es tief verwurzelten Intuitionen über die Endlichkeit menschlichen Lebens entspricht. Nicht nur die Gesellschaft, sondern auch Angehörige und Betroffene selbst bewerten einen Sterbefall bzw. drohenden Tod in jungen Jahren anders als in einer späten Lebensphase. Das zugrunde liegende Prinzip, also die Erfassung des Verlusts von Lebenszeit als Schaden, gilt jedoch in jedem Alter, also auch jenseits von willkürlichen Referenzaltern oder Lebenserwartungen zum Zeitpunkt der Geburt. Das ist ein starkes Argument für die Anwendung von Sterbetafelmethoden. Dabei sollten für Zwecke der nationalen Gesundheitsplanung die YLL konsequent auf Basis nationaler Sterbetafeln ermittelt werden. Die Verwendung internationaler Standards, die im Kontext der vergleichenden Gesundheitsberichterstattung unter dem Gesichtspunkt der Egalität angemessen ist, ist für den rein nationalen Zweck suboptimal. Dieser wird umso besser erreicht, je genauer der Indikator die Verhältnisse im Zielstaat abbildet.

Im Unterschied zur Berechnung von YLL auf der Basis der regulären nationalen Sterbetafeln hat die hier vorgeschlagene Variante der Sterbetafelmethode den Vorteil, dass sie bei der Berechnung ursachenspezifischer YLL den Gedanken der hypothetischen Elimination der jeweiligen Todesursache logisch konsistent umsetzt. Zwar sind die Unterschiede zur konventionellen Sterbetafelmethode bei den meisten Todesursachen gering, da sich die Elimination seltenerer Todesursachen auf die Lebenserwartung kaum auswirkt. Bei häufigen Todesursachen sind sie jedoch erheblich. Das zeigt sich zum Beispiel an der relativen Bedeutung der Herz-Kreislauf-Erkrankungen. Gemessen an den CELT-basierten YLL verursachten sie 2018 einen Verlust an Lebenszeit, der 90 % der durch Krebs verlorenen Lebensjahr entspricht. Gemessen an den herkömmlich berechneten EYLL beträgt dieser Wert nur 82 %. Bei einigen Erkrankungen kommt es zu einem Wechsel der Rangpositionen (Ränge 5/6 und 10/11).

Bei Anwendung der hier vorgeschlagenen Methode sind einige Punkte zu bedenken, die teils allgemeinerer, teils spezifischerer Art sind. In dieser Reihenfolge sind zu nennen:

Die Daten der Todesursachenstatistik gelten als nicht sehr valide [30, 31]. Die bekannten Schwächen belasten auch die hier verwendeten Zahlen der GBD-Studie mit Unsicherheit.

Wir haben Daten der GBD-Studie verwendet, da dort das Problem nichtinformativer Codes auf weithin akzeptierte Weise gelöst wurde. Todesursachenbereinigte Sterbetafeln und damit auch CELT-basierte YLL können methodisch analog auch mit den Originaldaten der Todesursachenstatistik berechnet werden. In diesem Fall werden auf der Ebene der Dreisteller gemäß ICD-10 „Sonstige ungenau oder nicht näher bezeichnete Todesursachen“ (ICD-10 Code R99) großes Gewicht bekommen. In der Onlinedatenbank der Bundesgesundheitsberichterstattung waren sie 2018 neunthäufigste Todesursache und für 6 % der verlorenen Lebensjahre unter 65 Jahre verantwortlich [7]. Ferner sind die im Methodenteil erwähnten Inkongruenzen der GBD- und ICD-Klassifikationen zu bedenken. Den möglichen Einfluss von Veränderungen der Codierpraxis beim Übergang von ICD‑9 zu ICD-10 schätzen wir aufgrund der Transformation in das spezifische Schema des GBD-Projekts als gering ein.

CELT-basierte YLL können wie herkömmlich berechnete verlorene Lebensjahre auch als rohe und altersstandardisierte Raten angegeben werden. Dies empfiehlt sich, wenn unterschiedlich große Populationen bzw. Populationen mit unterschiedlicher Altersstruktur verglichen werden sollen *und* bei dem Vergleich demografische Einflüsse „herausgerechnet“ werden sollen. Für die Priorisierung von Versorgungsbedarfen ist es allerdings angemessener, rohe Zahlen zu berichten, da demografiebedingte Bedarfsänderungen andernfalls verdeckt werden.

Zur Berechnung „verlorener gesunder Lebensjahre“ (Disability-adjusted Life Years, DALY) können CELT-basierte YLL wie üblich mit Jahren krankheitsbedingt reduzierter Lebensqualität kombiniert werden [32].

Die Methode beruht auf der Annahme, dass eine Person, die nicht an der Indexerkrankung stirbt, hinsichtlich der übrigen Erkrankungen ein durchschnittliches Sterberisiko hat. Die Annahme unabhängiger Sterberisiken ist nicht sehr realistisch [20, 33]. Wer an einer chronischen Erkrankung des Atmungssystems (zumeist chronisch-obstruktive Lungenerkrankung, COPD) stirbt, hatte wahrscheinlich auch ein erhöhtes Risiko für einen Tod infolge anderer Erkrankungen (insbesondere Herz-Kreislauf-Erkrankungen). Umgekehrt mag jemand, der an Hautkrebs stirbt, hinsichtlich anderer Todesursachen ein geringeres Risiko gehabt haben, da das Hautkrebsrisiko mit dem sozioökonomischen Status steigt [34–36], der wiederum positiv mit der Lebenserwartung korreliert [37]. Die Bedenken gelten allerdings für jedes Verfahren zur Berechnung todesursachenspezifischer verlorener Lebensjahre. Da die Bestimmung etwaiger Abhängigkeiten Daten über prävalente Erkrankungen zum Todeszeitpunkt voraussetzt und mit vielen Unsicherheiten behaftet ist, wird die Unabhängigkeit der Todesursachen im Allgemeinen als gegeben angenommen [38].

Die auf der Basis von ursachenbereinigten Sterbetafeln berechneten YLL haben eine gewöhnungsbedürftige Eigenschaft. Sie sind – ebenso wie die Gewinne an Lebenserwartung (s. oben) – nicht additiv, d. h., die CELT-basierten YLL durch Tod infolge Ursache x oder y sind nicht identisch mit der Summe aus CELT-basierten YLL durch Todesursache x und entsprechenden YLL durch Todesursache y. Wären sie additiv, ließe sich eine Zahl von Lebensjahren angeben, die durch Sterbefälle jedweder Ursache verloren gehen (oder – spiegelbildlich – gewonnen würden, wenn sämtliche Todesursachen eliminiert würden). Das ist aber offensichtlich keine sinnvolle Aussage. Um die Gesamtsterblichkeit abzubilden, steht der bewährte und einfache Indikator „Lebenserwartung bei Geburt“ zur Verfügung.

In der exemplarischen Anwendung der Methode auf die Todesursachen 1998 und 2018 werden Veränderungen sichtbar, die zumindest teilweise auf die Alterung der Gesellschaft zurückzuführen sind. Todesursachen mit relativ niedrigem durchschnittlichen Sterbealter (absichtliche Selbst- und Fremdverletzungen, Verletzungen im Straßenverkehr) verlieren an Bedeutung; Erkrankungen mit höherem durchschnittlichen Sterbealter (neurologische Störungen, Diabetes und Nierenerkrankungen) werden wichtiger. Demografische Veränderungen können jedoch nicht erklären, warum Krebs (durchschnittliches Sterbealter 1998: 71,5 Jahre) die Herz-Kreislauf-Erkrankungen (durchschnittliches Sterbealter 1998: 79,4 Jahre) als bedeutsamste Todesursache verdrängt hat. Hauptgrund könnte der zunehmende Einsatz leitliniengerechter Präventionsmaßnahmen und Therapien der koronaren Herzkrankheit sein [39]. Ob sich der langfristige Trend fortsetzt, ist unsicher. Es gibt Hinweise, dass sich die positive Entwicklung bei der kardiovaskulären Mortalität in den hochentwickelten Ländern abschwächt [40], während vielversprechende Innovationen in der Krebsbekämpfung noch am Anfang stehen. Soweit die neuen Ansätze letale Verläufe nicht verhindern, aber die Überlebenszeit verlängern, würde dies Effekte auf die Zahl der verlorenen Lebensjahre haben, die Zahl der Todesfälle aber nicht verringern.

## Fazit

Verlorene Lebensjahre sind ein in Deutschland wenig verbreiteter, aber aussagekräftiger Indikator zur Beschreibung todesursachenspezifischer Sterblichkeit. Die Wahl der jeweils besten Berechnungsweise hängt von sozialethischen Grundannahmen und vom Verwendungszusammenhang ab. Für die Steuerung der Gesundheitsversorgung ist es sinnvoll, verlorene Lebensjahre auf der Basis der tatsächlichen regionalen Lebenserwartungen zu berechnen, anstatt Referenzalter zu nutzen, wie sie bei internationalen Vergleichen üblich sind. Die hier vorgeschlagene Methode unter Verwendung todesursachenbereinigter Sterbetafeln vermeidet Inkonsistenzen herkömmlicher Strebetafelmethoden und führt insbesondere bei häufigen Todesursachen zu abweichenden Ergebnissen. Der Rechenaufwand ist zwar größer. Der konzeptionelle Schwierigkeitsgrad ist jedoch kaum höher als bei dem etablierten Indikator „Gewinn an Lebenserwartung“. Im Übrigen gilt, was für alle todesursachenspezifischen verlorenen Lebensjahre gilt: Ihre Validität kann verbessert werden, indem Abhängigkeiten ätiologisch konkurrierender Todesursachen in Rechnung gestellt werden [41, 42]. Die Aufgabe ist jedoch mit Daten einer monokausalen Todesursachenstatistik nicht lösbar und muss weiteren Untersuchungen vorbehalten bleiben.

## Supplementary Information




