# Supplementary material for: Years of life lost: known methods and a refined approach using the example of the most frequent causes of death in Germany
Source: Bundesgesundheitsblatt Gesundheitsforschung Gesundheitsschutz. 2021 Oct 1;64(11):1463–72. [Article in German] doi: 10.1007/s00103-021-03424-8 (PMC8485316; doi:10.1007/s00103-021-03424-8)
Supplement: Supplementary file 1 [file 103_2021_3424_MOESM1_ESM.pdf]

Bundesgesundheitsblatt 11/2021

Onlinematerial zum Beitrag:

Verlorene Lebensjahre: Bekanntes und Neues zur Methodik am Beispiel der häufigsten Todesursachen in Deutschland

Joachim Hübner[1], Johann Mattutat[2], Alexander Katalinic[1,2]

[1] Institut für Sozialmedizin und Epidemiologie, Universität zu Lübeck, Lübeck, Deutschland
[2] Institut für Krebsepidemiologie, Universität zu Lübeck, Lübeck, Deutschland

Korrespondenzadresse
Dr. med. Dr. jur. Joachim Hübner
Institut für Sozialmedizin und Epidemiologie
Universität zu Lübeck
Ratzeburger Allee 160
23562 Lübeck
Deutschland
joachim.huebner@uksh.de

Inhalt

Anhang 1: **Definition der untersuchten Todesursachen entsprechend der GBD-Studie**
Anhang 2: **Methodik der Berechnung von YLL auf der Basis von todesursachenbereinigten Sterbetafeln (CELT)**

Anhang 1: **Definition der untersuchten Todesursachen entsprechend der GBD-Studie**

Todesursache im GBD-Projekt	ICD-10	ICD-9
**Krebs**	C00-C13.9, C15-C25.9, C30-C34.9, C37-C38.8, C40-C41.9, C43-C45.9, C47-C54.9, C56-C57.8, C58-C58.0, C60-C68.8, C64-C67.9, C68.0-C68.8, C69-C75.8, C81-C86.6, C88-C96.9, D00.1-D00.2, D01.0-D01.3, D02.0-D02.3, D03-D06.9, D07.0-D07.2, D07.4-D07.5, D09.0, D09.2-D09.3, D09.8, D10.0-D10.7, D11-D12.9, D13.0-D13.7, D14.0-D14.3, D15-D16.9, D22-D24.9, D26.0-D27.9, D28.0-D28.1, D28.7, D29.0-D29.8, D30.0-D30.8, D31-D36, D36.1-D36.7, D37.1-D37.5, D38.0-D38.5, D39.1-D39.2, D39.8, D40.0-D40.8, D41.0-D41.8, D42-D43.9, D44.0-D44.8, D45-D47.9, D48.0-D48.6, D49.2-D49.4, D49.6, K62.0-K62.1, K63.5, N60-N60.9, N84.0-N84.1, N87-N87.9	140-148.9, 150-158.9, 160-164.9, 170-175.9, 180-183.8, 184.0-184.4, 184.8, 185-186.9, 187.1-187.8, 188-188.9, 189.0-189.8, 190-194.8, 200-208.9, 209.0-209.1, 209.4-209.5, 210.0-210.9, 211.0-211.8, 212.0-212.8, 213-213.9, 217-217.8, 219.0, 220-220.9, 221.0-221.8, 222.0-222.8, 223.0-223.8, 224-228.9, 229.0, 229.8, 230.1-230.8, 231.0-231.2, 232-232.9, 233.0-233.2, 233.4-233.5, 233.7, 234.0-234.8, 235.0, 235.4, 235.6-235.8, 236.1-236.2, 236.4-236.5, 236.7, 237-237.3, 237.5-237.9, 238.0-238.9, 239.2-239.4, 239.6, 569.0, 610-610.9, 622.1-622.2, 622.7
**Herz-Kreislauf-Erkrankungen**	B33.2, G45-G46.8, I01-I01.9, I02.0, I05-I09.9, I11-I11.9, I20-I25.9, I28-I28.8, I30-I31.1, I31.8-I37.8, I38-I41.9, I42.1-I42.8, I43-I43.9, I47-I48.9, I51.0-I51.4, I60-I63.9, I65-I66.9, I67.0-I67.3, I67.5-I67.6, I68.0-I68.2, I69.0-I69.3, I70.2-I70.8, I71-I73.9, I77-I83.9, I86-I89.0, I89.9, I98, K75.1	036.4, 391-391.9, 392.0, 393-398.9, 402-402.9, 410-414.9, 417-417.9, 420-423, 423.1-423.9, 424.0-424.9, 425.0-425.3, 425.5, 425.7-425.8, 427-427.3, 427.6-427.8, 429.0, 430-435.9, 437.0-437.2, 437.5-437.8, 440.2, 440.4, 441-443.9, 447-454.9, 456, 456.3-457, 457.1, 457.8-457.9, 459, 459.1-459.3
**Neurologische Störungen**	F00-F03.9, G10-G13.8, G20-G20.9, G23-G24, G24.1-G25.0, G25.2-G25.3, G25.5, G25.8-G26.0, G30-G31.1, G31.8-G31.9, G35-G37.9, G40-G41.9, G61-G61.9, G70-G72, G72.2-G73.7, G90-G90.9, G95-G95.9, M33-M33.9	290-290.9, 294.1-294.9, 330-331.2, 331.5-332.0, 333-337.9, 340-341.9, 345-345.9, 349, 349.2-349.8, 353.6-353.9, 356-356.9, 357.0-357.1, 357.3-357.4, 357.7, 358-359.9, 775.2
**Gastroenterologische Erkrankungen**	B18-B18.9, I84-I85.9, I98.2, K20-K29.9, K31-K31.8, K35-K38.9, K40-K42.9, K44-K46.9, K50-K52.9, K55-K62, K62.2-K62.6, K62.8-K62.9, K64-K64.9, K66.8, K67, K68-K68.9, K70-K70.3, K71.7, K74-K74.9, K75.2, K75.4-K76.2, K76.4-K77, K77.8, K80-K83.9, K85-K86.9, K90-K90.9, K92.8, K93.8, M09.1	455-455.9, 456.0-456.2, 530-536.1, 537-537.6, 537.8, 538, 540-543.9, 550-551.1, 551.3-552.1, 552.3-553.6, 555-558.9, 560-560.3, 560.8-560.9, 562-562.1, 564-564.1, 564.5-564.7, 565-566.9, 569.1-569.5, 569.7, 571-571.9, 572.2-573.0, 573.4-577.9, 579-579.2, 579.4-579.9, 787.1
**Chronische Erkrankungen des Atmungssystems**	D86-D86.2, D86.9, G47.3, J30-J35.9, J37-J39.9, J41-J46.9, J60-J63.8, J65-J68.9, J70, J70.8-J70.9, J82, J84-J84.9, J91-J92.9	135-135.9, 136.6, 327.2-327.8, 470, 470.9-474.9, 476-476.1, 477-479, 491-493.9, 495-504.9, 506-506.9, 508-509, 515, 516-517.8, 518.6, 518.9, 519.1-519.4
**Diabetes and Nierenerkrankungen**	D63.1, E10-E11.9, I12-I13.9, N00-N08.8, N15.0, N18-N18.9, P70.2, Q61-Q62.8	403-404.9, 580-583.9, 585-585.9, 589-589.9, 753-753.3, 775.1
**Absichtliche Selbst- und Fremdverletzungen**	U00-U03, X60-X64.9, X66-Y08.9, Y35-Y38.9, Y87.0-Y87.1, Y89.0-Y89.1	E950-E979, E990-E999
**Unabsichtliche Verletzungen**	L55-L55.9, L56.3, L56.8-L56.9, L58-L58.9, W00-W46.2, W49-W62.9, W64-W70.9, W73-W75.9, W77-W81.9, W83-W94.9, W97.9, W99-X06.9, X08-X39.9, X46-X48.9, X50-X54.9, X57-X58.9, Y40-Y84.9, Y88-Y88.3	349.0-349.1, 457.0, E856-E857, E861-E865, E867-E869, E870-E876, E878-E879, E880-E886, E888-E928, E930-E949
**Infektionen des Atmungssystems und Tuberkulose**	A10-A14, A15-A19.9, A48.1, A70, B90-B90.9, B97.4-B97.6, H70-H70.9, J00-J02.8, J03-J03.8, J04-J04.2, J05-J05.1, J06.0-J06.8, J09-J15.8, J16-J16.9, J20-J21.9, J36-J36.0, K67.3, K93.0, M49.0, N74.1, P23.0-P23.4, P37.0, U04-U04.9, U84.3	010-019.9, 034.0, 079.6, 137-137.9, 138.0-138.9, 381-383.9, 460-464.4, 464.8-464.9, 465.0-465.8, 466-469, 470.0, 475-475.9, 476.9, 480-482.8, 483.0-483.9, 484.1-484.2, 484.6-484.7, 487-489, 730.4-730.6
**Andere nichtübertragbare Erkrankungen**	D25-D26, D28.2, D52.1, D55-D58.9, D59.0-D59.3, D59.5-D59.6, D60-D61.9, D64.0, D66-D67, D68.0-D69.8, D70-D75.8, D76-D78.8, D86.8, D89-D89.3, E03-E07.1, E09-E09.9, E15.0, E16.0-E16.9, E20-E34.8, E36-E36.8, E65-E68, E70-E85.2, E88-E89.9, G24.0, G25.1, G25.4, G25.6-G25.7, G72.0, G93.7, G97-G97.9, I95.2-I95.3, I97-I97.9, I98.9, J70.0-J70.5, J95-J95.9, K43-K43.9, K62.7, K91-K91.9, K94-K95.8, M87.1, N10-N12.9, N14-N15, N15.1-N16.8, N20-N23.0, N25-N28.1, N29-N32.0, N32.3-N32.4, N34-N34.3, N36-N36.9, N39-N39.2, N41-N41.9, N44-N44.0, N45-N45.9, N49-N49.9, N65-N65.1, N72-N72.0, N75-N77.8, N80-N81.9, N83-N83.9, N99-N99.9, P96.0, P96.2, P96.5, Q00-Q07.9, Q10.4-Q18.9, Q20-Q28.9, Q30-Q36, Q37-Q45.9, Q50-Q60.6, Q63-Q86, Q86.1-Q87.8, Q89-Q89.8, Q90-Q93.9, Q95-Q99.8, R50.2, R95-R95.9	218-219, 219.1-219.9, 236.0, 240-243.9, 244.0-244.1, 244.3-244.8, 245-246.9, 251-259.9, 270-273.9, 275-276, 277-277.2, 277.4-277.9, 278.0-278.8, 282-284.9, 286-286.5, 286.7-289.7, 357.6, 518.7, 519.0, 536.4, 539-539.9, 551.2, 552.2, 564.2-564.4, 569.6, 579.3, 588-588.9, 590-590.9, 592-593.8, 594-599.6, 599.8, 601-602.9, 604-604.9, 608.2, 617-618.9, 620-620.9, 621.4-621.9, 622.3-622.6, 629-629.8, 740-749.0, 749.2-752.9, 753.4-758.9, 759.0-759.8, 775.3, 779.4-779.5, 788.0, 798-798.0
**Substanzbezogene Störungen**	F10-F16.9, F18-F19.9, G31.2, G72.1, P04.3-P04.4, P96.1, Q86.0, R78.0-R78.5, X45-X45.9, X65-X65.9, Y15-Y15.9	291-292.9, 303-303.9, 304.0-304.8, 305-305.9, 357.5, 760.7, 790.3, E850, E860
**Verletzungen im Straßenverkehr**	V00-V86.9, V87.2-V87.3, V88.2-V88.3, V90-V98.8	E800-E807, E830-E838, E840-E849
**Schwangerschaftsassoziierte und neonatale Störungen**	N96, N98-N98.9, O00-O07.9, O09-O16.9, O20-O26.9, O28-O36.9, O40-O48.1, O60-O77.9, O80-O92.7, O96-O98.6, O98.8-P04.2, P04.5-P05.9, P07-P15.9, P19-P22.9, P24-P29.9, P36-P36.9, P38-P39.9, P50-P61.9, P70-P70.1, P70.3-P72.9, P74-P78.9, P80-P81.9, P83-P84, P90-P94.9, P96, P96.3-P96.4, P96.8	630-636.9, 638-638.9, 640-679.1, 760-760.6, 760.8-768, 768.2-770, 770.1-771, 771.4-775.0, 775.4-779.3, 779.6-779.8

Quelle: Institute for Health Metrics and Evaluation (IHME). Global Burden of Disease Study 2017 (GBD 2017) Causes of Death and Nonfatal Causes Mapped to ICD Codes.

Anhang 2: **Methodik der Berechnung von YLL auf der Basis von todesursachenbereinigten Sterbetafeln (CELT)**

Es sei i eine Erkrankung mit zu berechnender Krankheitslast und es sei die Bevölkerung aufgeteilt in Altersgruppen $t_{1,\dots,21}$ von $0-1, 1-4, 5-9, 10-14, 15-19, \dots, 85-89, 90-94$ und $95+$ Jahren. Ferner sei I_t die Bevölkerungszahl in der jeweiligen Altersgruppe im Referenzjahr, d_t die Anzahl der Todesfälle in der entsprechenden Altersgruppe und d_t^i die Anzahl der Todesfälle attributabel zu i.

Es sei $r_t^i = \frac{d_t^i}{d_t}$ der Anteil an Todesfällen attributabel zu i.[1] Ferner sei $p_t = \frac{I_t - d_t}{I_t}$ die beobachtete Überlebenswahrscheinlichkeit in Altersgruppe t und $p_t^i = p_t^{1-r_t^i}$ die nach Arias et al. (2013) um i bereinigte Überlebenswahrscheinlichkeit. Die nach Periodenansatz berechnete um i bereinigte Wahrscheinlichkeit des Überlebens der gesamten Altersgruppe ist entsprechend $P_t^i = (p_t^i)^k$ mit einem Exponenten $\{k = 1 \text{ für } t_1;\ k = 4 \text{ für } t_2;\ k = 5 \text{ sonst}\}$. Für das obere Ende der Sterbetafel sei $P_{t_{21}}^i = 0$ und eine maximale Lebenserwartung von 100 Jahren angenommen.

Von einer Referenz-Population von $I_{t_1}^{i,begin} = 100.000$ leben am Ende der Altersgruppe t_j noch $I_{t_j}^{i,end} = I_{t_1}^{i,begin} \cdot \prod_{x=1}^{j} P_{t_x}^i$ Individuen für $j = 1 \dots 21$. Da das Ende einer Altersperiode dem Beginn der nachfolgenden entspricht, gilt gleichzeitig $I_{t_j}^{i,end} = I_{t_{j+1}}^{i,begin}$ für $j = 1, \dots, 20$. Die Anzahl der in der Altersgruppe t durchlebten Lebensjahre entspricht $L_t^i = I_t^{i,end} \cdot k + \left(I_t^{i,begin} - I_t^{i,end}\right) \cdot m$ für die Periodenlänge $\{k = 1 \text{ für } t_1;\ k = 4 \text{ für } t_2;\ k = 5 \text{ sonst}\}$ und die erwartete Überlebensdauer m der in dieser Altersgruppe verstorbenen. Als Sterbezeitpunkt wurde jeweils die Mitte der betroffenen Altersperiode angenommen. Abweichend wurde für die Gruppe der Neugeborenen die durchschnittliche Überlebensdauer der Verstorbenen aus den Sterbetafeln von DeStatis übernommen, sodass sich $\{m = 48/356 \text{ für } t_1;\ m = 2 \text{ für } t_2;\ m = 2{,}5 \text{ sonst}\}$ ergibt. Die Gesamtzahl der zu Beginn der Altersgruppe t noch zu durchlebenden Lebensjahre ist aufsummiert $T_{t_j}^i = \sum_{x=21-j}^{21} L_{t_x}^i$ für $j = 1, \dots, 21$. Je Individuum verbleiben zu Beginn der Altersgruppe t folglich $e_t^i = \frac{T_t^i}{I_t^{i,begin}}$ erwartete Lebensjahre. Für jeden Todesfall in Altersgruppe t attributabel zu i verliert man entsprechend $l_{t_j}^i = \frac{e_{t_j}^i + e_{t_{j+1}}^i}{2}$ für $j = 1, \dots, 21$ mit $e_{t_{22}}^i = 0$. Aufsummiert ergibt dies das Endergebnis $Lost^i = \sum_{x=1}^{21} d_{t_x}^i \cdot l_{t_x}^i$.

---

[1] Arias et al. (2013) nennen an dieser Stelle zusätzlich eine Korrektur für Sterbefälle mit unbekanntem Alter. Da solche Fälle in den GBD-Daten nicht auftreten, vereinfacht sich der Korrekturterm zum Faktor 1 und wurde hier gekürzt.
